# Diagnostic Accuracy of Female Pelvic Ultrasonography in Differentiating Precocious Puberty From Premature Thelarche: A Systematic Review and Meta-analysis

**DOI:** 10.3389/fendo.2021.735875

**Published:** 2021-09-01

**Authors:** Nam Nhat Nguyen, Linh Ba Phuong Huynh, Minh Duc Do, Tien Yun Yang, Meng-Che Tsai, Yang-Ching Chen

**Affiliations:** ^1^International Ph.D. Program in Medicine, College of Medicine, Taipei Medical University, Taipei, Taiwan; ^2^Ph.D. Program in School of Nutrition and Health Sciences, College of Nutrition, Taipei Medical University, Taipei, Taiwan; ^3^Center for Molecular Biomedicine, University of Medicine and Pharmacy at Ho Chi Minh City, Ho Chi Minh City, Vietnam; ^4^School of Medicine, College of Medicine, Taipei Medical University, Taipei, Taiwan; ^5^Division of Genetics, Endocrinology, and Metabolism, Department of Pediatrics, National Cheng Kung University Hospital, Tainan, Taiwan; ^6^College of Medicine, National Cheng Kung University, Tainan, Taiwan; ^7^Department of Family Medicine, Taipei Medical University Hospital, Taipei, Taiwan; ^8^Department of Family Medicine, School of Medicine, College of Medicine, Taipei Medical University, Taipei, Taiwan

**Keywords:** precocious puberty, premature thelarche, pelvic ultrasonography, uterine length, diagnostic accuracy

## Abstract

**Background:**

The gonadotropin-releasing hormone (GnRH) stimulation test is the benchmark for diagnosing precocious puberty (PP). However, it is invasive, time-consuming, costly, and may create an unpleasant experience for participants. Moreover, some overlaps may occur between PP and premature thelarche (PT) in the early stage of PP. Female pelvic ultrasonography may provide additional information to help differentiate PP from PT and subsequently initiate early treatment. In this study, we aimed to first directly compare pelvic ultrasonography parameters between PP and PT groups and secondly, investigate their diagnostic accuracy compared with the GnRH stimulation test.

**Methods:**

A systematic search of the PubMed/MEDLINE, EMBASE, Scopus, and Cochrane Library databases was performed up to March 31, 2021. All types of studies, except for case reports and review articles, were included. The GnRH stimulation test was used to confirm PP diagnosis. Those whose organic conditions might cause PP were excluded. The mean, standard deviation, sensitivity, and specificity of each parameter were documented. Forest plots were constructed to display the estimated standardized mean differences (SMDs) from each included study and the overall calculations. A bivariate model was used to calculate the pooled sensitivity, specificity, positive likelihood ratio (PLR), negative likelihood ratio (NLR), and diagnostic odds ratio (DOR).

**Results:**

A total of 13 studies were included for analysis. The SMDs (95% confidence interval – CI) in ovarian volume, fundal-cervical ratio, uterine length, uterine cross-sectional area, and uterine volume between PP and PT groups were 1.12 (0.78–1.45; p < 0.01), 0.90 (0.07–1.73; p = 0.03), 1.38 (0.99–1.78; p < 0.01), 1.06 (0.61–1.50; p < 0.01), and 1.21 (0.84–1.58; p <0.01), respectively. A uterine length of 3.20 cm yielded a pooled sensitivity of 81.8% (95% CI 78.3%–84.9%), specificity of 82.0% (95% CI 61.0%–93.0%), PLR of 4.56 (95% CI 2.15–9.69), NLR of 0.26 (95% CI 0.17–0.39), and DOR of 19.62 (95% CI 6.45–59.68). The area under the summary receiver operating characteristics curve was 0.82.

**Conclusion:**

Female pelvic ultrasonography may serve as a complementary tool to the GnRH stimulation test in differentiating PP from PT.

**Systematic Review Registration:**

https://www.crd.york.ac.uk/prospero/display_record.php?ID=CRD42021232427, ID: CRD42021232427.

## Introduction

Untreated precocious puberty (PP) may result in numerous adverse outcomes (1–6). Correct identification and early initiation of appropriate treatment for PP using gonadotropin-releasing hormone (GnRH) analogs in cases of central PP might limit these adverse outcomes. For clarification, PP refers to the central type of PP throughout this paper.

Diagnostic challenges exist regarding the identification of PP and discrimination between PP and other variants of puberty, including premature thelarche (PT). Available clinical manifestations or laboratory tests alone cannot be used to establish a definite PP diagnosis because of the multifactorial and multistage nature of puberty. Hormonal testing (i.e., the GnRH stimulation test) is often necessary for detecting hypothalamic-pituitary-gonadal (HPG) axis activation, which is a reliable indicator of puberty. Despite that, it is an invasive, time-consuming, and costly technique that may create an unpleasant experience for participants. Another major disadvantage of this test is its relatively low sensitivity despite its high specificity; this is primarily attributed to the inadequate luteinizing hormone (LH)-response to the GnRH in the initial stage of premature sexual development (7). Therefore, the diagnostic value of this hormonal test is limited.

PT is a benign condition involving isolated and non-progressive breast development in girls, which is often diagnosed by normal growth velocity and concordant bone age with chronological age, and does not require medical treatment (8). However, it may mimic early clinical manifestations of PP and thereby pose diagnostic challenges in equivocal cases. Studies have reported that approximately 9%–14% of PT cases were first misdiagnosed but finally confirmed as PP during follow-up (9, 10). This is because some overlaps may occur between PP and PT, even with the use of the GnRH stimulation test, especially in the early stage of PP (7).

Pelvic ultrasonography has been suggested to facilitate the differentiation of PP from PT because it is non-invasive, saves time, is affordable, and is widely used in clinical practice. Previously, it has been, however, primarily indicated to exclude organic causes of peripheral early puberty, such as ovarian cysts and tumors (11–13). Moreover, international consensus on the definite cutoffs for ultrasonography measurements in PP is unavailable. Although a previous consensus reported the helpfulness of pelvic ultrasonography in differentiating PP from PT, it also revealed that cutoff values for uterine length in children with PP might widely vary between 3.4 and 4.0 cm (14). In short, the optimal cutoff values remain a controversial topic.

This systematic review and meta-analysis aimed to first directly compare the pelvic ultrasonography parameters between PP and PT patients and secondly, determine the diagnostic accuracy of these parameters in comparison with the GnRH stimulation test.

## Methods

### Population, Indicator, Comparison, Outcomes, and Study Design

Participants include girls referred to the pediatric endocrinology departments due to appearance of secondary sexual characteristics before the age of 8 years old. The indicators were ultrasonography measurements on female pelvic ultrasonography. The GnRH stimulation test was considered the comparator (gold standard) to confirm PP diagnosis, after taking clinical manifestations and radiological assessment into account. Regarding the outcomes of the first aim, we performed a comparative meta-analysis to identify standardized mean differences (SMDs) between the PP and PT groups with respect to each selected parameter. For the second aim, we performed a diagnostic accuracy meta-analysis to calculate the pooled sensitivity and specificity.

All types of studies, except for case reports and review articles, were included. Those whose organic conditions might cause PP were excluded. These criteria were pre-outlined in the selected articles. The following parameters were included in the comparative analysis due to sufficient data: ovarian volume, fundal-cervical ratio (FCR), uterine length, uterine cross-sectional area (CSA), and uterine volume. Two independent reviewers completed the process of searching for, screening, reviewing, and extracting data. In case of disagreements between the reviewers, a third reviewer was consulted to reach a final decision. We used the Preferred Reporting Items for Systematic Reviews and Meta-Analyses (PRISMA) to verify the transparent reporting.

### Systematic Review Protocol

This systematic review and meta-analysis was registered in the PROSPERO International Prospective Register of Systematic Reviews (ID: CRD42021232427).

### Search Strategy and Data Sources

We systematically searched the PubMed/MEDLINE, EMBASE, Scopus, and Cochrane databases for relevant articles up to 31/03/2021; the search did not include restrictions on language or publication year. The following keywords were used in the search (**Table S1**): “precocious puberty,” “premature thelarche,” “ultrasound,” “sonography,” and “echography”. Furthermore, we identified additional relevant articles by manually searching the references of the articles found.

### Data Extraction

The mean, standard deviation of each parameter measurement, the number of observations in each group, and other demographic variables were documented. True positives, true negatives, false positives, and false negatives were directly extracted from the papers or indirectly calculated from sensitivity and specificity when appropriate. If the required data were not sufficiently furnished in an article, the corresponding author of that article was contacted through e-mail to request for the missing statistics.

### Data Analysis

The risk of bias of included studies in the comparative meta-analysis was assessed using the Newcastle–Ottawa Scale (15). On the other hand, we adopted a revised version of the Quality Assessment of Diagnostic Accuracy Studies (QUADAS-2) tool to examine the risk of bias and applicability concerns (16).

Forest plots were constructed to display the estimated SMDs from each included study and the overall calculations. Heterogeneity among studies was tested using Cochran’s Q test and I^2^. A random-effects model would be adopted when heterogeneity was observed between studies, as confirmed by a Cochran’s Q test p value of <0.1 or an I^2^ of >50%. Otherwise, a fixed-effects model was preferred. Meta-regression and subgroup analysis were performed to explore sources of heterogeneity if indicated. Publication bias for each parameter was examined using Egger’s test.

A bivariate model was used to calculate the pooled sensitivity, specificity, positive likelihood ratio (PLR), negative likelihood ratio (NLR), and diagnostic odds ratio (DOR), along with their corresponding 95% confidence intervals (CIs). The performance of pelvic ultrasonography parameters was represented by a summary receiver operating characteristics (SROC) curve and the area under the SROC curve (AUSROC). The closer the SROC curve is to the left corner and the higher AUSROC is, the higher discrimination ability of the test. The cutoff values of pooled sensitivity and specificity were defined using multiple thresholds modeling (17, 18).

An asymmetry test based on Deeks’ funnel plot was performed to validate the asymmetry assumption, with a p value of <0.1 signifying publication bias (19). Fagan’s nomogram was used to determine the posttest probability of PP after pelvic ultrasonography. A large-scale epidemiologic study in Korea reported that the overall prevalence of PP in girls was 0.4% (20). In the present study, we used this prevalence as the pretest probability of PP, and it was located on the left axis of the nomogram, whereas the PLR and NLR of pelvic ultrasonography parameters were located on the middle axis. These variables were used to project the posttest probability of PP, which was located on the right axis of the nomogram.

A two-sided p value of <0.05 was considered statistically significant. All analyses were performed using R software (version 4.0.2; R Foundation for Statistical Computing; Vienna, Austria).

## Results

### Literature Search and Study Selection

**Figure 1:**
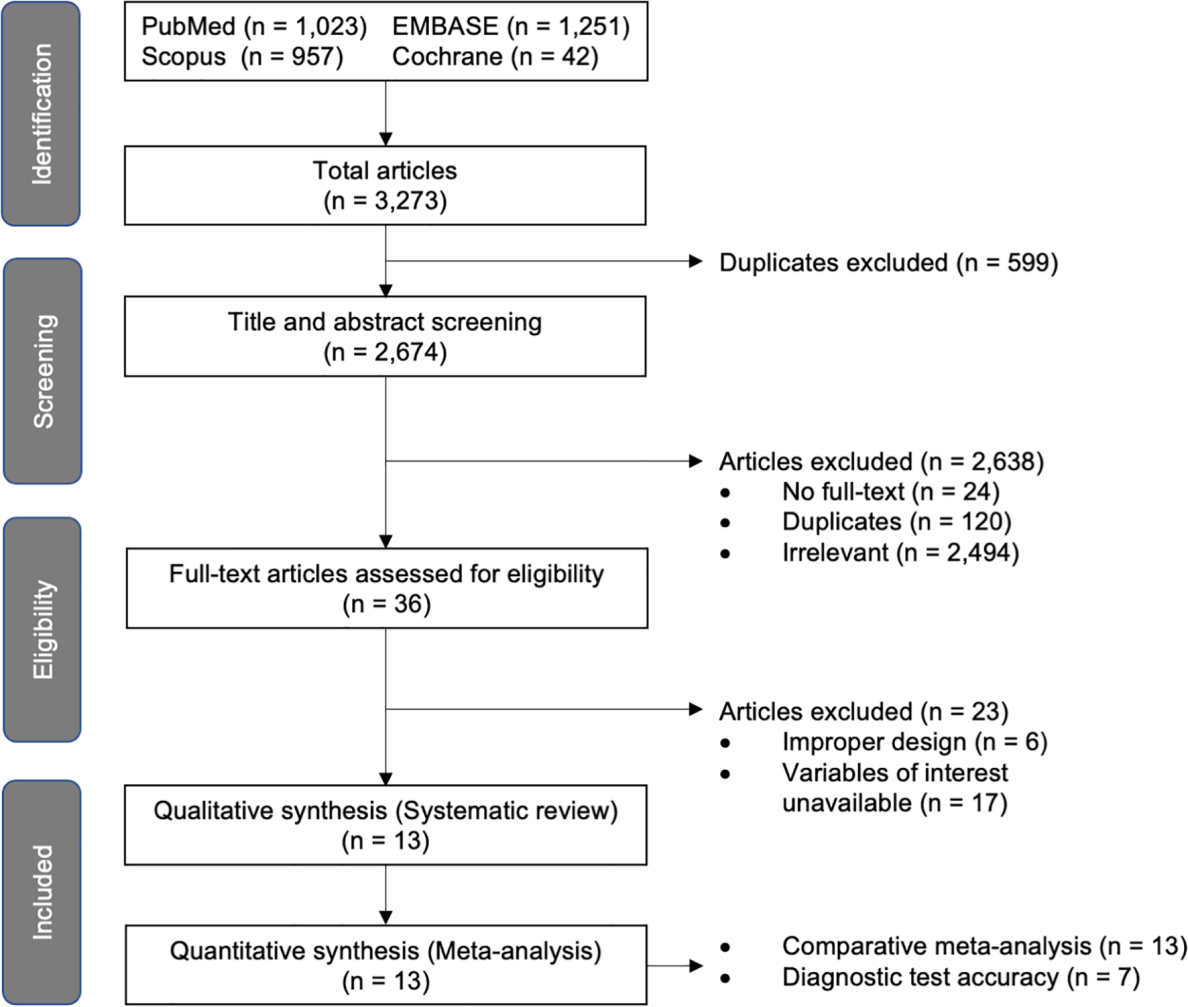
PRISMA flowchart for summarizing the study selection process.

A total of 3,273 articles were identified from the mentioned databases as shown in the PRISMA flowchart (**Figure 1**). After performing deduplication, we observed that 2,674 articles remained and screened their titles and abstracts. Of these articles, 2,638 were excluded due to full text unavailability (n = 20), duplication (n = 120), and irrelevancy (n = 2,494); thus 36 reports remained for full text review and eligibility assessment. Six of them were then excluded because they reported a combined group of premature thelarche and other puberty variants, while 17 others could not provide the outcomes of interest, even after we contacted the corresponding authors. No additional articles were found through the manual search. Finally, 13 studies were included in this systematic review and meta-analysis. They were all deemed suitable for the comparative analysis, and seven were deemed appropriate for the diagnostic accuracy analysis.

### Study and Participant Characteristics

A total of 1,977 subjects were available for analysis (**Table 1**). Among the selected studies in this review, 3 were retrospective studies (10–12), 8 were prospective studies (13, 21, 23–26, 28, 29), and 2 were cross-sectional studies (22, 27). All patients had been referred to outpatient clinics to evaluate early breast development and any other pubertal progression signs. In the GnRH stimulation test, participants with a peak LH value of >5 UI/L were considered to have PP (PP group), and those with a peak LH value of <5 IU/L were considered to have PT (PT group) (14). Pelvic ultrasonography was performed using a conventional full-bladder technique with 3–13.5 MHz transducers and was interpreted by skilled and trained physicians. During the computation of ovarian and uterine volumes, both the ovaries and the uteri were considered ellipses, as demonstrated by the following formula: V = longitudinal diameter (length) × transverse diameter × fundal anterior–posterior diameter × 0.5233 (29).

**Table 1:** Characteristics of the included studies (systematic review).

Study	Year	Country	Study design	N	Population	Main findings
Yuan et al. (21)	2020	China	Prospective	669	Girls with Tanner B2 breast development whose age of initiation was <8 years	Uterine length, uterine volume, and average ovarian volume were significantly greater in the PP group than in the PT group
Karaoglan et al. (11)	2018	Turkey	Retrospective	267	Girls aged <8 years who had breast development classified as at least Tanner B2	- Uterine length and ovarian volume were significantly higher among patients with PP
						- Sens/Spec: uterine length, 0.81/0.84; ovarian volume, 0.77/0.74
Yu et al. (12)	2015	South Korea	Retrospective	248	Girls aged 7–8 years who had breasts with Tanner stage 2 or higher	- Patients with PP who had normal weight had significantly greater uterine length, uterine CSA, and uterine volume. FCR and ovarian volume of both normal-weight and obese patient did not differ between the two groups
						- Sens/Spec: uterine length, 0.83/0.34; uterine CSA, 0.50/0.78; uterine volume, 0.59/0.70
Bizzarri et al. (10)	2014	Sweden	Retrospective	91	Girls referred for thelarche before the age of 3 years	- Uterine length was significantly greater in the PP group than in the PT group
						- Sens/Spec: uterine length, 0.67/0.93
Binay et al. (22)	2014	Turkey	Cross-sectional	100	Girls with suspected onset of breast budding before the age of 8 years	- Uterine length, FCR, and ovarian volume were significantly larger in the PP group than in the PT group
						- Sens/Spec: uterine length, 0.93/0.87; FCR, 0.92/0.87; ovarian volume, 0.73/0.90
Kilic et al. (13)	2012	Turkey	Prospective	184	Girls who had signs of PP before the age of 8 years	Uterine and ovarian sizes of the PP group were statistically higher than those of the PT group
Eksioglu et al. (23)	2012	Turkey	Prospective	87	Girls aged <8 years who presented with breast development	All pelvic ultrasonography measurements in the PP group were significantly higher, except for ovarian volume, than in the PT group
Badouraki et al. (24)	2008	Greece	Prospective	47	Girls referred for examination due to apparent breast development before the age of 8 years	- All pelvic ultrasonography measurements were significantly larger in the PP for both subgroups (0–6 years) and (>6–8 years)
						- Sens/Spec: uterine length, 0.83/0.91; uterine volume, 0.88/0.74; FCR, 0.79/0.74; ovarian volume, 0.79/0.70
de Vries et al. (25)	2006	Israel	Prospective	103	Girls referred for evaluation due to the appearance of breast buds between ages 4 and 8 years	- All of the uterine and ovarian measurements were significantly different between the PP and PT groups, except for ovarian volume, which was at borderline
						- Sens/Spec: uterine length, 0.80/0.58; uterine volume, 0.89/0.89
Battaglia et al. (26)	2003	Italy	Prospective	32	Girls referred for the evaluation of premature breast development before the age of 8 years	- Uterine and ovarian sizes were larger in the PP group than in the PT group
						- Sens/Spec: uterine volume, 0.88/1.0
Herter et al. (27)	2002	Brazil	Cross-sectional	16	Girls aged 1–7 years were diagnosed as having PP or PT	Uterine length, uterine volume, uterine area, and ovarian volume of girls with PT were different from those of girls with PP.
Buzi et al. (28)	1998	United Kingdom	Prospective	67	Girls who presented with breast enlargement before 7 years of age or whose other secondary sexual characteristics appeared before age 8 years	Uterine length, uterine CSA, and ovarian volume but not FCR were significantly greater in the PP group than in the PT group
Haber et al. (29)	1995	Germany	Prospective	66	Girls with isolated PT or progression of pubertal signs, accelerated growth velocity and advanced bone age	- Ultrasonography measurements of the uterus and ovaries could differentiate PP from PT
						- Sens/Spec: uterine length, 0.9/1.0; uterine volume, 1.0/1.0; ovarian volume, 0.82/0.95

*CSA, cross-sectional area; FCR, fundal-cervical ratio; N, number of participants; PP, precocious puberty; PT, premature thelarche; Sens, sensitivity; Spec, specificity.

### Risk of Bias Assessment

The Newcastle–Ottawa assessment results revealed that all of the studies were rated as “Good” or “Fair” (**Table S2**). Moreover, seven studies included in the diagnostic test accuracy meta-analysis yielded acceptable risks of bias using the QUADAS-2 tool (**Table S3**).

### Synthesized Findings

#### Comparative Analysis of Ultrasonography Parameters Between PP and PT Patients

**Figure 2:**
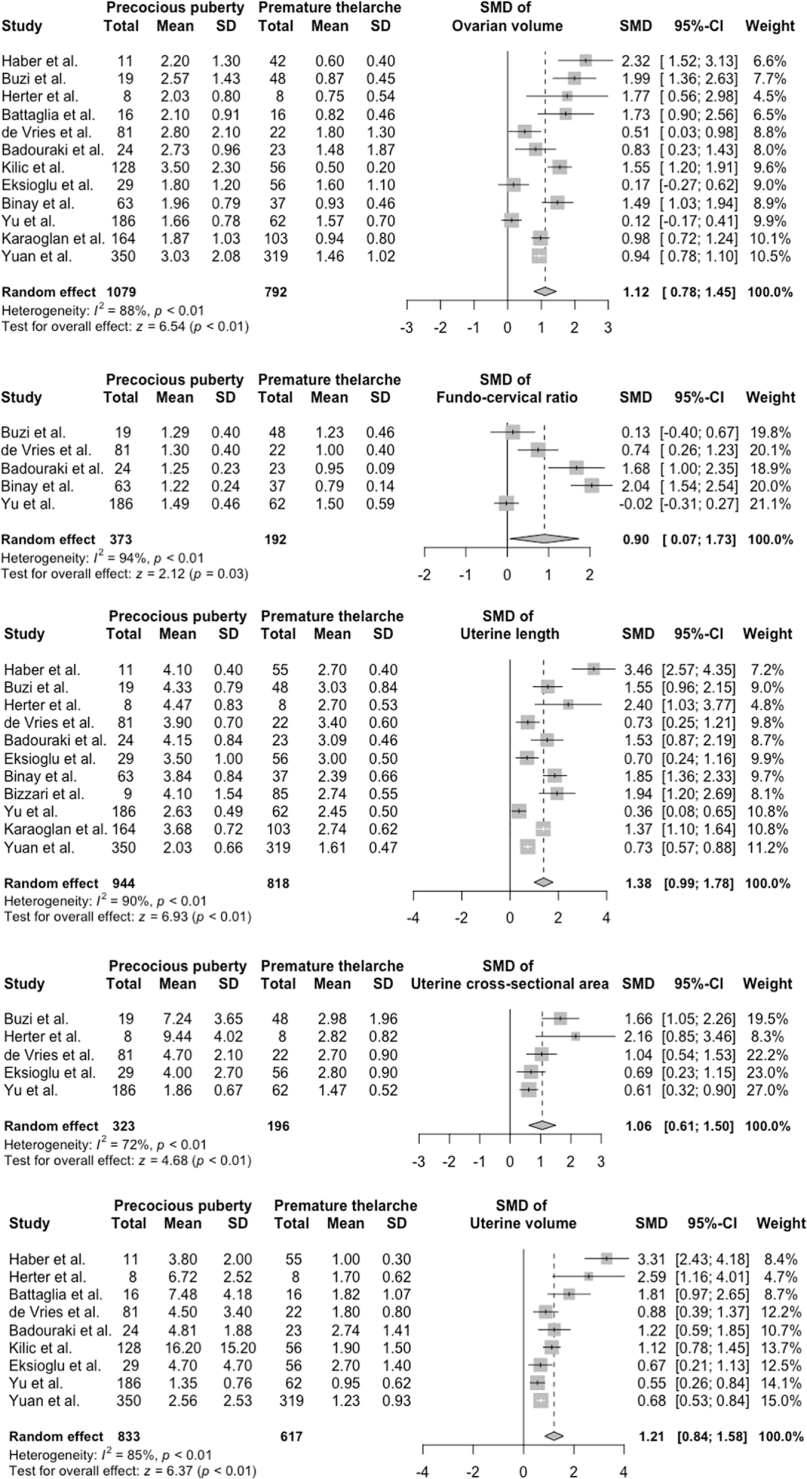
Forest plots of standardized mean difference in ultrasonography parameters between PP and PT groups. PP, precocious puberty; PT, premature thelarche; SD, standard deviation; SMD, standardized mean difference.

Heterogeneity tests revealed that the Q test p value was <0.1 and that the I^2^ statistic was >50%. Accordingly, we used a random-effects model to calculate the pooled effect sizes of each parameter. The overall SMDs (95% CI) in ovarian volume, FCR, uterine length, uterine CSA, and uterine volume between the PP and PT groups were 1.12 (0.78–1.45; p < 0.01), 0.90 (0.07–1.73; p = 0.03), 1.38 (0.99–1.78; p < 0.01), 1.06 (0.61–1.50; p < 0.01), and 1.21 (0.84–1.58; p < 0.01), respectively. These results were visualized using forest plots (**Figure 2**).

#### Publication Bias

Egger’s tests revealed possible publication biases for uterine length (p = 0.02) and uterine volume (p = 0.02; **Table S4**). The trim-and-fill method was then performed for these two parameters. No significant differences between pre- and after-filling effect sizes were found (p = 0.25 for uterine length and p = 0.06 for uterine volume).

#### Meta-Regression and Subgroup Analysis

**Figure 3:**
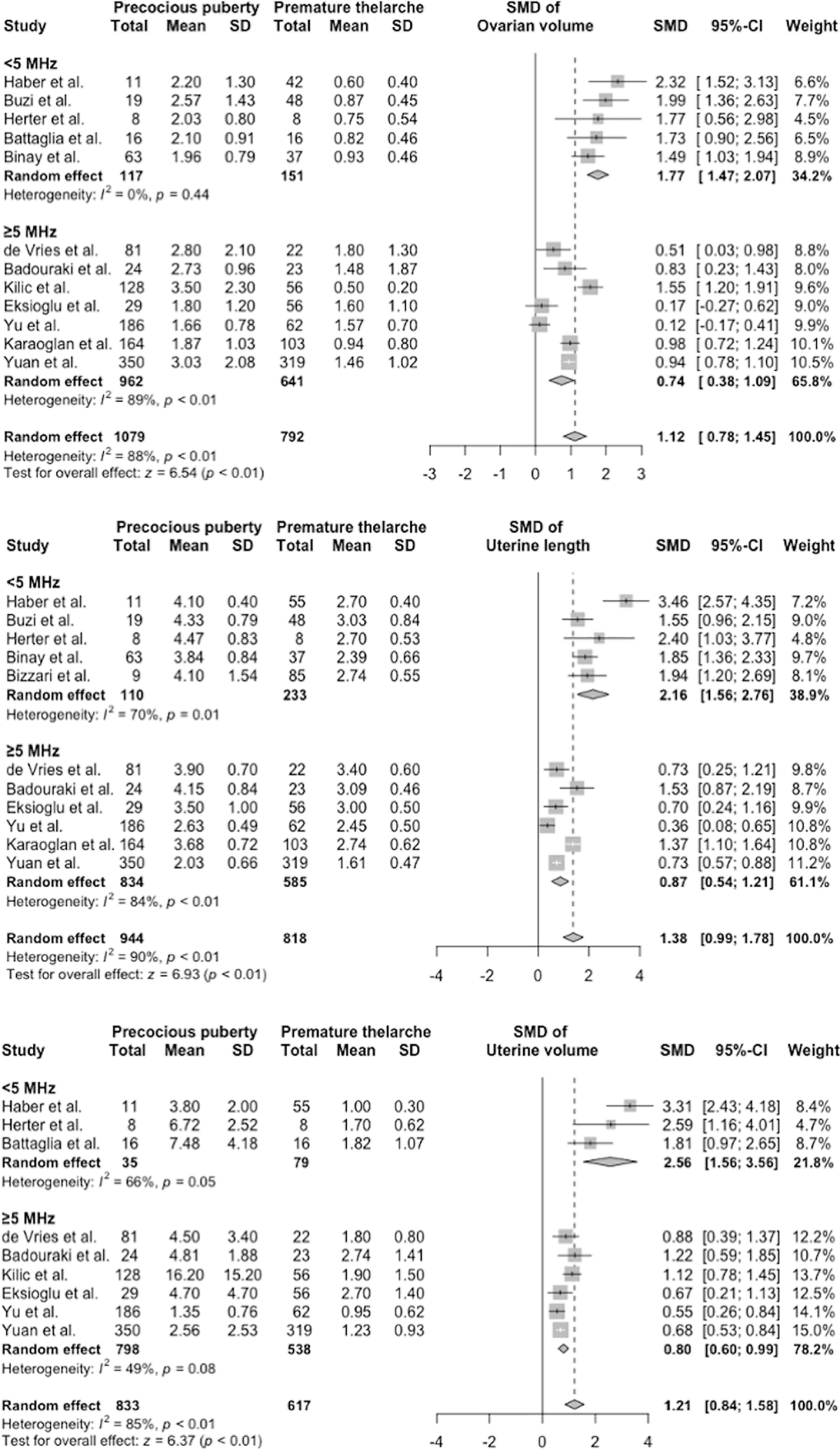
Subgroup analysis of ovarian volume, uterine length, and uterine volume. CI, confidence interval; SD, standard deviation; SMD, standardized mean difference.

Meta-regression and subgroup analysis identified the probe frequency as the main culprit that affected ultrasonography measurements, followed by the publication year and chronological age at referral (**Table S5** and **Figure 3**). Studies using low frequency probes tended to produce higher SMDs than those using higher frequency probes. After subgrouping parameters with sufficient data based on the mean probe frequency, the I^2^ in group 1 (<5 MHz) and group 2 (≥5 MHz) shrank moderately (**Figure 3**). Test for subgroup differences revealed statistically significant differences between group 1 and group 2 (all p < 0.001).

#### Diagnostic Accuracy of Ultrasonography Parameters Compared to the GnRH Stimulation Test

**Figure 4:**
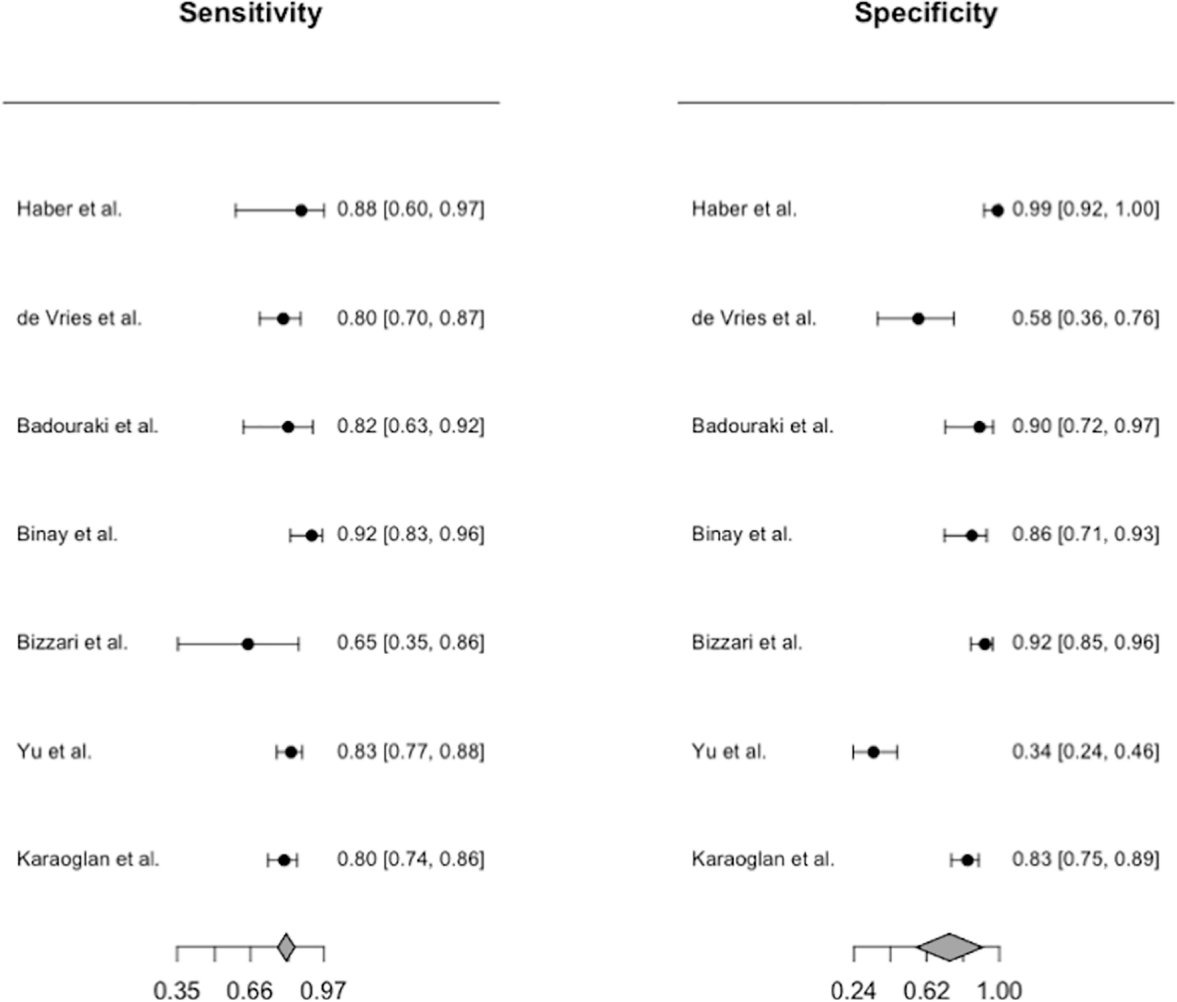
Forest plots for the pooled diagnostic estimates of sensitivity and specificity of uterine length.

**Figure 5:**
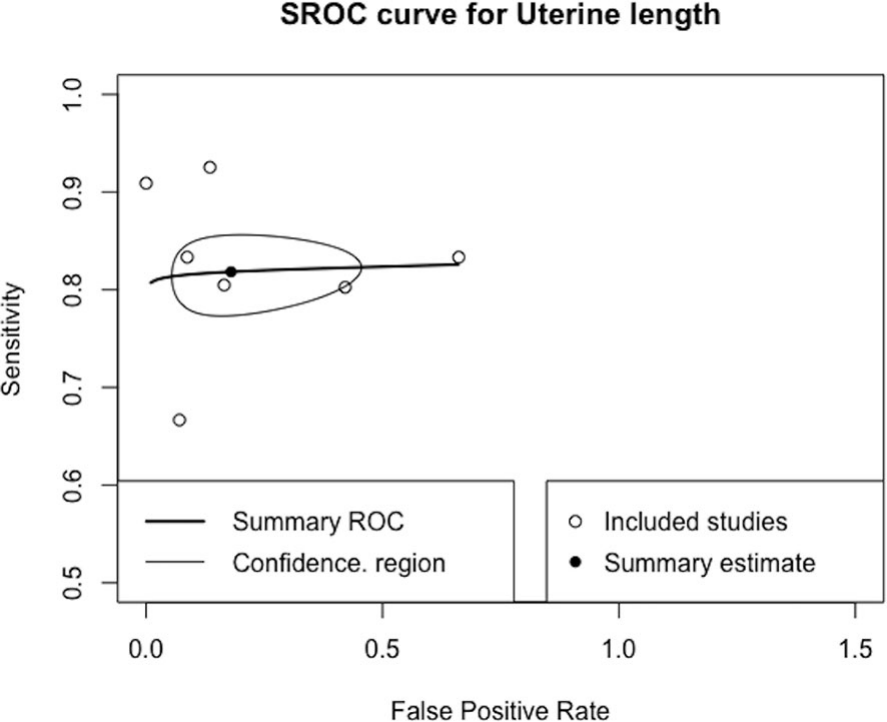
SROC curve of uterine length. SROC, summary receiver operating characteristic.

**Figure 6:**
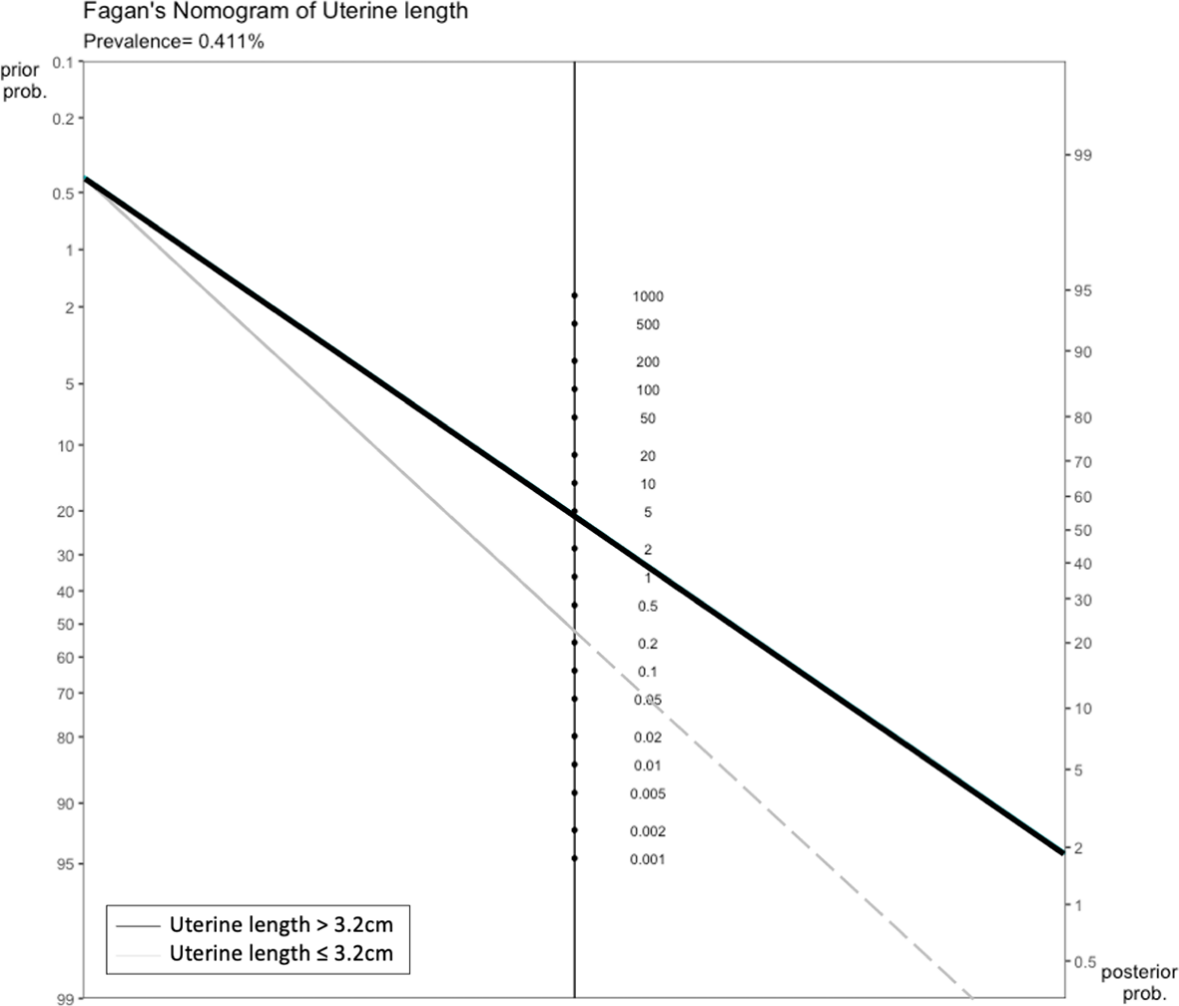
Fagan’s nomogram of uterine length.

Only the uterine length had sufficient data for diagnostic accuracy analysis. After combining the reports, we observed a pooled sensitivity of 81.8% (95% CI 78.3%–84.9%), specificity of 82% (95% CI 61%–93%) (**Figure 4**), PLR of 4.56 (95% CI 2.15–9.69), NLR of 0.26 (95% CI 0.17–0.39), and DOR of 19.62 (95% CI 6.45–59.68). These sensitivity and specificity values were equivalent to a cutoff value of 3.2 cm for uterine length, as determined from the multiple-thresholds modeling (18). The heterogeneity revealed that the Q-test p value was 0.32 and that the I^2^ statistic was 14.2%, indicating that the estimates were consistent among the included studies. **Figure 5** displayed the SROC curve of uterine length with an AUSROC of 0.8, suggesting acceptable discrimination between PP and PT. No clear publication bias could be identified from the asymmetry test based on Deeks’ funnel plot (p = 0.34, **Figure S1**). As mentioned, the projected posttest probabilities of PP using the nomogram were 1.9% and 0.1%, respectively (**Figure 6**). In other words, girls referred to pediatric clinics with a uterine length >3.2 cm had a 1.9% probability of having PP, whereas those with a uterine length of ≤3.2 cm only had a 0.1% chance of having PP. Meanwhile, this rate in overall population without knowing ultrasonography results was approximately 0.4% (20).

## Discussion

### Summary of Main Findings

This systematic review and meta-analysis confirmed that pelvic ultrasonography was an appropriate diagnostic tool to differentiate PP from PT. All investigated ultrasonography parameters were significantly greater in the PP group than in the PT group. The early increases in uterine and ovarian sizes represent the estrogenic effects of HPG axis activation on internal female genitalia, which indicates PP. In our meta-analysis, uterine length, CSA, and volume were determined to be valuable markers for differentiating PP from PT. The uterine length of 3.2 cm exhibited satisfactory diagnostic accuracy as indicated by sensitivity and specificity levels of 81.8% and 82.0%, respectively (AUSROC 0.82). It could be readily interpreted from our Fagan’s nomogram that for suspected cases referred to clinics due to breast development before the age of 8 years, a girl who has a uterine length of >3.2 cm would confer an approximately 17-time greater risk of PP than one having a uterine length of ≤3.2 cm. It is thus reasonably recommended that the ultrasonography should be performed during the initial evaluation of PP to help clinicians recognized those with high probability of PP.

The FCR was also determined to be a valuable indicator of puberty. In mid-childhood, the anteroposterior diameter of the uterine fundus and cervix are nearly the same, resulting in an FCR of ≤1. After puberty onset, the fundus widens under hormonal effects relative to the cervix, increasing the FCR to >1. However, previous studies have yielded inconsistent results; for example, some have reported a significantly higher FCR in PP (22, 24, 27), whereas others have not (12, 28). After pooling these studies, we found a meaningful difference in FCR between the PP and PT groups, suggesting that this parameter could successfully differentiate PP from PT. Nevertheless, the FCR might not be reliable in patients aged >7 years because Herter et al. reported that the FCR could not differentiate between different forms of early puberty in this age group (30).

Ovarian parameters are generally inferior PP markers compared with uterine parameters, as confirmed by several studies (12, 23, 25). This is partly because the shape of the ovaries is asymmetrical instead of perfectly oval; therefore pelvic ultrasonography’s results, particularly through the transabdominal approach, are challenging to interpret. Furthermore, the ovarian volume remains relatively constant from birth to puberty; this engenders considerable challenges in differentially diagnosing PT right before the pubertal onset, a period during which the volume in individuals with PP may overlap with that in those with PT (aged approximately seven years) (31). Finally, the ovaries begin to increase in size, in addition to exhibiting other pubertal signs, approximately two years later than the uterus does (31, 32). Thus, this explains the lower sensitivity of ovarian parameters in the early identification of PP compared with uterine parameters.

Numerous pelvic ultrasonography parameters had been suggested to help diagnose PP, including ovarian morphology, quantity of large follicles, maximum follicular diameter, uterine endometrial echogenicity, endometrial thickness, uterine arterial impedance, and vaginal wall thickness. However, none of these parameters have been proven to be reliable indicators of correct pubertal stages and HPG axis activity. Moreover, data on these parameters are insufficient for a meta-analysis because various studies have adopted different definitions and classifications of the parameters. Accordingly, additional studies are warranted to confirm the diagnostic values of these parameters.

### Strengths and Limitations

According to our literature review, this study is the first to establish an explicit pooled cutoff for uterine length for differentiating PP from PT. Our findings are expected to provide clinicians with a more comprehensive perspective that can help them enhance PP diagnostic accuracy in equivocal cases and determine which patients need treatment. Furthermore, our findings reveal the potential application of several ultrasonography parameters other than uterine length. Accordingly, pelvic ultrasonography could become a complementary diagnostic tool to the GnRH stimulation test. The trim-and-fill result implies that even in the presence of publication bias due to missing studies, our pooled standardized mean differences still reflected true effect size.

Despite covering an appealing topic, our study has some limitations. First, we could include only observational studies rather than randomized controlled trials. However, a large number of girls with early pubertal development were analyzed in this multicenter review, and all of the studies were rated as “Good” or “Fair” in the risk-of-bias assessment and produced an acceptable result when being combined. Second, we found a high degree of heterogeneity among the studies, which could be attributed to unrecognized confounders. Nevertheless, this finding reflects real-world scenarios upon which most pediatric endocrinologists must rely. In these circumstances, the ultrasonography parameters vary by chronological age, abdominal fat mass, degree of bladder fullness, presence of dilated intestinal loops, uterine position, and ultrasonographic equipment resolution. Although studies using low frequency probes tended to have higher SMDs that was not as expected from our general knowledge, they had relatively smaller sample sizes and wider 95% CIs. Therefore, we documented this result but also further suggested that it should be interpreted cautiously. Regarding publication year, ultrasonography equipment with higher probe frequency and higher resolution has been advancing over time. Therefore, the significant result of publication year resulted from meta-regression might be attributable to the difference in probe frequency. Chronological age was negatively associated with effect sizes in meta-regression of uterine length and uterine volume, suggesting that the later these participants were referred to clinics, the smaller differences in uterine parameters were found between PP and PT groups. This was because PT girls tended to approach their normal puberty after following up, thus narrowing the gap between the two groups. In our analysis, chronological age at referral was comparable between two groups in most of the studies. However, even in the presence of age difference between two individuals, these parameters could still be useful in differentiating PP from PT. In more detail, there was a limited progression in ultrasonography parameters from birth to puberty. In other words, ultrasonography measurements are stable or only increase modestly during childhood until the HPG axis activation exerts estrogenic effects on genitalia. It can be seen that such an age difference will not affect the ultrasonography results much unless one actually suffers from PP. Therefore, our meta-analysis findings are relevant to the routine clinical contexts and could be used as a reference during the diagnostic workup for children with suspected PP or PT.

### Conclusion

To conclude, girls with PP had significantly greater uterine and ovarian measurements as determined by pelvic ultrasonography than did those with PT. Furthermore, uterine length represented a reliable marker to differentiate PP from PT, thereby reducing the possibility of misdiagnosing PP. Therefore, pelvic ultrasonography emphasizing these measurements should be considered as an adjunct to clinical examination, bone radiography, and laboratory tests to enhance diagnostic precision.

## Data Availability Statement

The original contributions presented in the study are included in the article/**Supplementary Material**. Further inquiries can be directed to the corresponding authors.

## Author Contributions

NN conceived of the original idea. NN and LH performed systematic searching, study selection, quality assessment, data extraction, and meta-analysis with help from TYY. Y-CC and M-CT verified the analytical methods and supervised the findings of this work. M-CT and MD gave clinically relevant advice. NN wrote the manuscript with support from Y-CC and M-CT. Y-CC supervised the project. All authors contributed to the article and approved the submitted version.

## Funding

All phases of this study were supported by the Ministry of Science and Technology, Taiwan, grant 110-2628-B-038-014-.

## Conflict of Interest

The authors declare that the research was conducted in the absence of any commercial or financial relationships that could be construed as a potential conflict of interest.

## Publisher’s Note

All claims expressed in this article are solely those of the authors and do not necessarily represent those of their affiliated organizations, or those of the publisher, the editors and the reviewers. Any product that may be evaluated in this article, or claim that may be made by its manufacturer, is not guaranteed or endorsed by the publisher.

## Abbreviations

AUSROC, Area under the summary receiver operating characteristic curve; CSA, Cross-sectional area; DOR, Diagnostic odds ratio; FCR, Fundal-cervical ratio; GnRH, Gonadotropin-releasing hormone; HPG, Hypothalamic-pituitary-gonadal; NLR, Negative likelihood ratio; PLR, Positive likelihood ratio; PP, Precocious puberty; PT, Premature thelarche; SROC, Summary receiver operating characteristic curve.
