# Supplementary material for: Diagnostic Accuracy of Female Pelvic Ultrasonography in Differentiating Precocious Puberty From Premature Thelarche: A Systematic Review and Meta-analysis
Source: Front Endocrinol (Lausanne). 2021 Sep 1;12:735875. doi: 10.3389/fendo.2021.735875 (PMC8442957; doi:10.3389/fendo.2021.735875)
Supplement: Supplementary file 1 [file DataSheet_1.docx]

**Supplementary Information**

**Table S1.** Search strategy (all fields included)

| **PubMed** | | |
| --- | --- | --- |
| **No** | **Query** | **Results** |
| #1 | (Precocious puberty) OR (early puberty) OR (premature thelarche) | 12,307 |
| #2 | Ultrasound OR sonography OR echography | 1,685,110 |
| #3 | #1 AND #2 | 1,023 |
| **EMBASE** | | |
| #1 | (Precocious puberty) OR (early puberty) OR (premature thelarche) | 18,409 |
| #2 | Ultrasound OR sonography OR echography | 768,724 |
| #3 | #1 AND #2 | 1,251 |
| **Scopus** | | |
| #1 | (Precocious puberty) OR (early puberty) OR (premature thelarche) | 78,446 |
| #2 | (Pelvic ultrasound) OR (Pelvic sonography) OR (Pelvic echography) | 65,330 |
| #3 | Female OR women OR girls | 13,055,909 |
| #4 | #1 AND #2 AND #3 | 957 |
| **Cochrane** | | |
| #1 | (Precocious puberty) OR (early puberty) OR (premature thelarche) | 109 |
| #2 | Ultrasound OR sonography OR echography | 1,152 |
| #3 | #1 AND #2 | 42 |

**Table S2.** Newcastle–Ottawa Quality Assessment Scale for comparative studies

| **Study** | **Selection** | **Comparability** | **Outcome** | **Overall quality** |
| --- | --- | --- | --- | --- |
| Yuan et al. 2020 | ★★★ | ★ | ★★ | Fair |
| Karaoglan et al. 2018 | ★★ | ★★ | ★★★ | Fair |
| Yu et al. 2015 | ★★ | ★★ | ★★ | Fair |
| Bizzari et al. 2014 | ★★ | ★★ | ★★★ | Fair |
| Binay et al. 2014 | ★★ | ★★ | ★★ | Fair |
| Kilic et al. 2012 | ★★★ | ★★ | ★★★ | Good |
| Eksioglu e al. 2012 | ★★★ | ★ | ★★★ | Good |
| Badouraki e al. 2008 | ★★★ | ★★ | ★★★ | Good |
| de Vries et al. 2006 | ★★★ | ★★ | ★★★ | Good |
| Battaglia et al. 2003 | ★★★ | ★ | ★★★ | Good |
| Herter et al. 2002 | ★★ | ★★ | ★★ | Fair |
| Buzi et al. 1998 | ★★ | ★ | ★★ | Fair |
| Haber et al. 1995 | ★★ | ★ | ★★★ | Fair |

**Table S3.** QUADAS-2 tool for diagnostic test accuracy studies

| **Study** | **Risk of bias** | | | | **Applicability concerns** | | |
| --- | --- | --- | --- | --- | --- | --- | --- |
|  | **Patient selection** | **Uterine length** | **GnRH test** | **Flow & timing** | **Patient selection** | **Uterine length** | **GnRH test** |
| Karaoglan et al. | ☺ | ☹ | ☺ | ☺ | ☺ | ☺ | ☺ |
| Yu et al. | ? | ☺ | ☺ | ☺ | ☺ | ☺ | ☺ |
| Bizzari et al. | ☺ | ☹ | ☺ | ? | ☹ | ? | ☺ |
| Binay et al. | ? | ☺ | ☺ | ☺ | ☺ | ☺ | ☺ |
| Badouraki et al. | ☺ | ☺ | ☺ | ☺ | ☺ | ☺ | ☹ |
| de Vries et al. | ☺ | ☺ | ☺ | ☹ | ☺ | ☺ | ☺ |
| Haber et al. | ☺ | ☺ | ☺ | ☺ | ☺ | ☺ | ☹ |

☺ Low risk ☹ High risk ? Unclear risk

**Table S4.** Analysis of publication bias by Egger’s test

| **Parameters** | **Intercept** | **SE** | **Slope** | **t-test** | **df** | **P-value** |
| --- | --- | --- | --- | --- | --- | --- |
| Ovarian volume | 1.81 | 1.61 | 0.65 | 1.12 | 10 | 0.29 |
| Fundo-cervical ratio | 8.50 | 4.89 | -1.22 | 1.74 | 3 | 0.18 |
| Uterine length | 3.70 | 1.35 | 0.39 | 2.73 | 9 | 0.02 |
| Uterine cross-sectional area | 3.72 | 1.24 | 0.06 | 3.00 | 3 | 0.06 |
| Uterine volume | 3.30 | 1.07 | 0.34 | 3.08 | 7 | 0.02 |

*SE, standard error; df, degree of freedom

**Table S5.** Meta-regression analysis on potentially confounding factors

| **Parameters** | **Confounding factors** | **Estimate** | **SE** | **P-value** |
| --- | --- | --- | --- | --- |
| Ovarian volume | Publication (year) | -0.05 | 0.02 | 0.04 |
|  | Sample size (n) | -0.00 | 0.00 | 0.39 |
|  | Region (Europe) | -0.63 | 0.41 | 0.16 |
|  | Study design (cohort) | 0.54 | 0.59 | 0.39 |
|  | Probe frequency (≥5 MHz) | -1.10 | 0.29 | 0.003 |
|  | Chronological age (years old) | -0.29 | 0.13 | 0.06 |
|  | Bone age (years old) | -0.13 | 0.46 | 0.81 |
|  | Age at first signs (years old) | -0.45 | 0.44 | 0.41 |
|  | BMI SDS | 1.34 | 0.55 | 0.13 |
| Fundo-cervical ratio | Publication (year) | 0.04 | 0.07 | 0.60 |
|  | Sample size (n) | -0.01 | 0.01 | 0.35 |
|  | Region (Europe) | -0.93 | 0.80 | 0.33 |
|  | Study design (cohort) | 1.44 | 0.84 | 0.19 |
|  | Probe frequency (≥5 MHz) | -0.32 | 0.95 | 0.76 |
|  | Chronological age (years old) | -0.58 | 0.41 | 0.29 |
|  | Bone age (years old) | -0.02 | 0.15 | 0.89 |
|  | Age at first signs (years old) | insufficient data for analysis | | |
|  | BMI SDS | 4.15 | 1.16 | 0.17 |
| Uterine length | Publication (year) | -0.06 | 0.03 | 0.05 |
|  | Sample size (n) | -0.00 | 0.00 | 0.17 |
|  | Region (Europe) | -0.85 | 0.49 | 0.12 |
|  | Study design (cohort) | 0.75 | 0.72 | 0.33 |
|  | Probe frequency (≥5 MHz) | -1.26 | 0.35 | 0.006 |
|  | Chronological age (years old) | -0.32 | 0.10 | 0.02 |
|  | Bone age (years old) | insufficient data for analysis | | |
|  | Age at first signs (years old) | -0.52 | 2.36 | 0.86 |
|  | BMI SDS | 2.92 | 1.26 | 0.26 |
| Uterine cross-sectional area | Publication (year) | -0.06 | 0.01 | 0.02 |
|  | Sample size (n) | -0.00 | 0.00 | 0.19 |
|  | Region (Europe) | -0.08 | 0.62 | 0.91 |
|  | Study design (cohort) | 1.21 | 0.82 | 0.24 |
|  | Probe frequency (≥5 MHz) | -1.03 | 0.28 | 0.04 |
|  | Chronological age (years old) | -0.22 | 0.19 | 0.37 |
|  | Bone age (years old) | insufficient data for analysis | | |
|  | Age at first signs (years old) | insufficient data for analysis | | |
|  | BMI SDS | insufficient data for analysis | | |
| Uterine volume | Publication (year) | -0.09 | 0.02 | 0.004 |
|  | Sample size (n) | -0.00 | 0.00 | 0.22 |
|  | Region (Europe) | -0.56 | 0.62 | 0.40 |
|  | Study design (cohort) | 1.38 | 1.16 | 0.27 |
|  | Probe frequency (≥5 MHz) | -1.73 | 0.37 | 0.002 |
|  | Chronological age (years old) | -0.45 | 0.11 | 0.006 |
|  | Bone age (years old) | -0.10 | 0.27 | 0.74 |
|  | Age at first signs (years old) | insufficient data for analysis | | |
|  | BMI SDS | 0.51 | 0.13 | 0.16 |

*BMI SDS, body-mass-index standard deviation score; SE, standard error


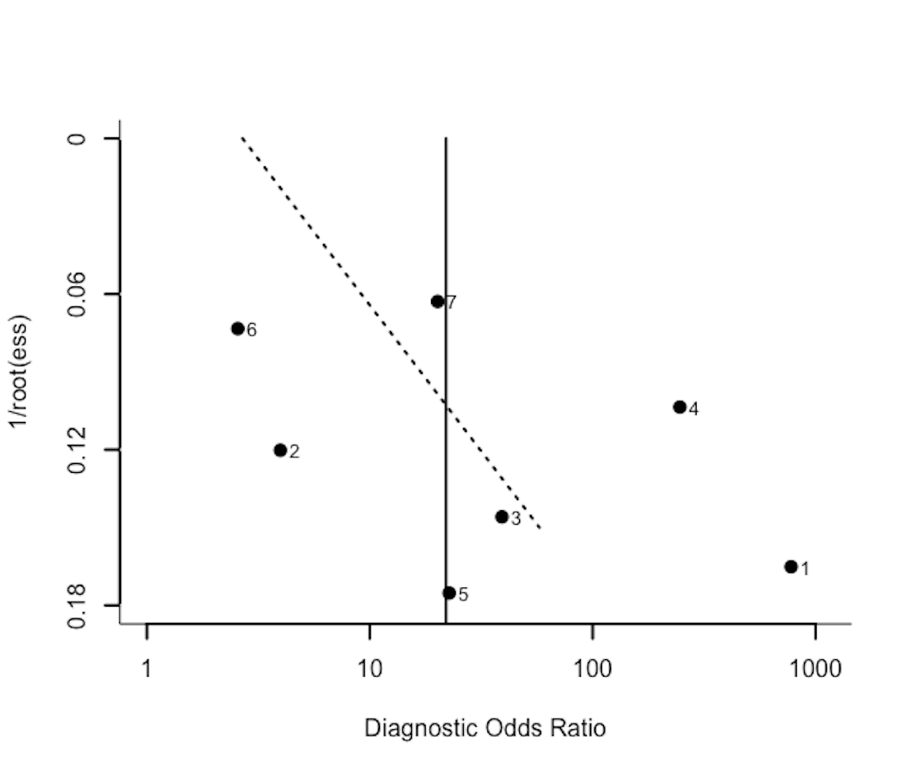


**Figure S1.** Deeks’ funnel plot for evaluating publication bias of diagnostic test accuracy studies. The y axis displayed the inverse of the square root of the effective sample size (1/root(ess)). The x axis displayed the diagnostic odds ratio (DOR). A p-value of 0.34 and a symmetrical funnel shape both indicated that publication bias was absent.
